# Supplementary material for: Changes in Attitudes towards Influenza and Pneumococcal Vaccination during the Subsiding COVID-19 Pandemic—Results of a Longitudinal Survey Study among Risk Groups in Germany between 2021 and 2023
Source: Vaccines (Basel). 2024 Sep 21;12(9):1080. doi: 10.3390/vaccines12091080 (PMC11435497; doi:10.3390/vaccines12091080)
Supplement: Supplementary file 1 [file vaccines-12-01080-s001.zip › vaccines-3192603-supplementary.pdf]

Supplement

Questionnaire

Demographics

1. How old are you?
2. What is your gender?
3. What is your highest level of education?
4. Are you currently suffering or have you suffered from cancer in the last 5 years?
5. What chronic diseases do you suffer from?

Vaccination knowledge

6. Which of the following vaccinations are recommended for people of your age and with any pre-existing medical conditions? *(True, False or I don't know.)*
    a. Vaccination against influenza viruses
    b. Vaccination against pneumococci (bacteria that can cause pneumonia, for example)
    c. Vaccination against the corona virus (Covid-19)

Vaccination attitude (Influenza/Pneumococci/COVID-19)

7. Please indicate to what extent you agree with the following statements.

    (1-strongly disagree, 2-disagree, 3-neutral, 4-agree, 5-strongly angry, I don't know)
    a. Vaccination against Influenza/Pneumococci/COVID-19 is necessary.
    b. Vaccination against Influenza/Pneumococci/COVID-19 is a good idea.
    c. Vaccination against Influenza/Pneumococci/COVID-19 is beneficial.

Risk perception (Influenza/Pneumococci/COVID-19)

8. How dangerous do you think the following pathogens are for you?

    (1-very low, 2-low, 3-moderate, 4-high, 5-very high, I don't know)
    a. Influenza virus
    b. Pneumococci (bacteria that can cause pneumonia, for example)
9. What do you think is the probability that you will contract the following pathogens if you are **not** vaccinated?

    (1-very low, 2-low, 3-moderate, 4-high, 5-very high, I don't know)
    a. Influenza virus
    b. Pneumococci (bacteria that can cause pneumonia, for example)
10. How dangerous do you think **vaccination** against the following pathogens is for you?

    (1-very low, 2-low, 3-moderate, 4-high, 5-very high, I don't know)
    a. Influenza virus
    b. Pneumococci (bacteria that can cause pneumonia, for example)
    c. Corona virus (COVID-19

11. How high do you estimate **the effectiveness of vaccination** against the following pathogens?

    (1-very low, 2-low, 3-moderate, 4-high, 5-very high, I don't know)

    a. Influenza virus
    b. Pneumococci (bacteria that can cause pneumonia, for example)
    c. Corona virus (COVID-19

Psychological antecedents of vaccination (5C)

(Subscale in brackets were not displayed to the participants)

12. Please indicate to what extent you agree with the following statements.

    (1-strongly disagree, 2-disagree, 3-neutral, 4-agree, 5-strongly angry, I don't know)

    a. I am completely confident that vaccines are safe. (Confidence)
    b. Vaccinations are effective. (Confidence)
    c. Regarding vaccines, I am confident that public authorities decide in the best interest of the community. (Confidence)
    d. Vaccination is unnecessary because vaccine preventable diseases are not common anymore. (Complacency)
    e. My immune system is so strong, it also protects me against diseases. (Complacency)
    f. Vaccine-preventable diseases are not so severe that I should get vaccinated. (Complacency)
    g. Everyday stress prevents me from getting vaccinated. (Constraints)
    h. For me, it is inconvenient to receive vaccinations. (Constraints)
    i. Visiting the doctor's makes me feel uncomfortable; this keeps me from getting vaccinated. (Constraints)
    j. When I think about getting vaccinated, I weight benefits and risks to make the best decision possible. (Calculation)
    k. For each vaccination, I closely consider whether it is useful for me. . (Calculation)
    l. It is important for me to fully understand the topic of vaccination, before I get vaccinated. . (Calculation)
    m. When everyone is vaccinated, I don't have to get vaccinated, too. [R] (Collective Responsibility)
    n. I get vaccinated because I can also protect people with a weaker immune system. (Collective Responsibility)
    o. Vaccination is a collective action to prevent the spread of diseases. (Collective Responsibility)

Table S1. Means of Vaccination Attitude, Risk Perception for COVID-19 and 5C Psychological Antecedents of Vaccination in 2021 and 2023.

	2021		2023		Change		
	M_1	SE	M_2	SE	$M_2 – M_1$	SE	p-value
COVID-19							
Danger of vaccination	2.455	0.042	2.210	0.046	-0.245	0.043	< 0.001*
Effectiveness of vaccination	3.970	0.035	3.679	0.049	-0.291	0.047	< 0.001*
Vaccination attitude	4.188	0.040	3.474	0.056	-0.714	0.052	< 0.001*
Confidence	3.689	0.035	3.499	0.039	-0.190	0.034	< 0.001*
Calculation	4.025	0.035	4.099	0.036	0.074	0.033	0.025*
Constraints	1.938	0.037	1.764	0.036	-0.174	0.029	< 0.001*
Complacency	2.211	0.032	2.105	0.033	-0.107	0.026	< 0.001*
Collective responsibility	3.657	0.039	3.660	0.042	0.003	0.031	0.922

Table S2. Reliability for the Vaccination Attitude Scales and the Subscales of the Psychological Antecedents of Vaccination Hesitancy (5C).

	**2021**	**2023**
	ω [95%-CI]	ω [95%-CI]
Vaccination Attitude		
Influenza	0.91 [0.90, 0.91]	0.93 [0.92, 0.95]
Pneumococci	0.92 [0.91, 0.94]	0.93 [0.92, 0.95]
COVID-19	0.93 [0.92, 0.94]	0.96 [0.95, 0.97]
Psychological Antecedents (5C)		
Confidence	0.84 [0.82, 0.86]	0.82 [0.78, 0.86]
Calculation	0.82 [0.79, 0.84]	0.78 [0.72, 0.82]
Constraints	0.73 [0.69, 0.77]	0.78 [0.92, 0.82]
Complacency	0.62 [0.56, 0.67]	0.58 [0.90, 0.72]
Collective responsibility	0.73 [0.69, 0.99]	0.78 [0.73, 0.82]

Note. ω = McDonald’s Omega; CI = Confidence interval.
